# Comparison of T1-mapping and T2-weighted imaging for diagnostic oedema assessment in ST-segment elevation myocardial infarction

**DOI:** 10.1186/1532-429X-18-S1-Q9

**Published:** 2016-01-27

**Authors:** Sheraz A Nazir, Abhishek Shetye, Jamal N Khan, Andrew P Vanezis, Anvesha Singh, Prathap Kanagala, Gerry P McCann

**Affiliations:** 1 grid.9918.90000000419368411University of Leicester, Leciester, United Kingdom; 2 grid.269014.80000000104359078University Hospitals of Leicester, Leicester, United Kingdom

## Background

Myocardial oedema (area-at-risk, AAR) is typically imaged using a pre-contrast T2-weighted short tau inversion recovery (T2w-STIR) sequence on cardiovascular magnetic resonance (CMR) imaging. However, this sequence is prone to motion and rhythm artefact, signal dropout, blood-pool artefact, surface coil signal inhomogeneity and potentially prohibitive long breath-hold duration. This susceptibility to artefacts limits utility of T2w-STIR in large clinical trials where attainment of diagnostic quality oedema imaging in the majority is necessary to determine myocardial salvage: a measure of reperfusion success and a strong predictor of adverse remodeling and prognosis post ST-segment elevation myocardial infarction (STEMI). We compare AAR quantified on T2w-STIR imaging with novel T1-mapping on 3.0T CMR post STEMI.

## Methods

Fifty-five patients underwent CMR 1-5 days following presentation with STEMI. AAR was quantified using semi-automatic thresholding on T2w-STIR images and resulting parametric colour maps from T1 Modified Look Locker Inversion Recovery (MOLLI) sequences performed with the patient breathing freely and with motion correction algorithm applied (MOCO-T1). Paired t-tests were used to compare AAR derived using the two sequences. Pearson's correlation coefficient was used to assess correlation. Inter-sequence agreement was assessed using the Bland-Altman method, coefficient of variation (CoV) and two-way mixed-effect intra-class correlation coefficient (ICC) for absolute agreement.

## Results

**Figure 1:**
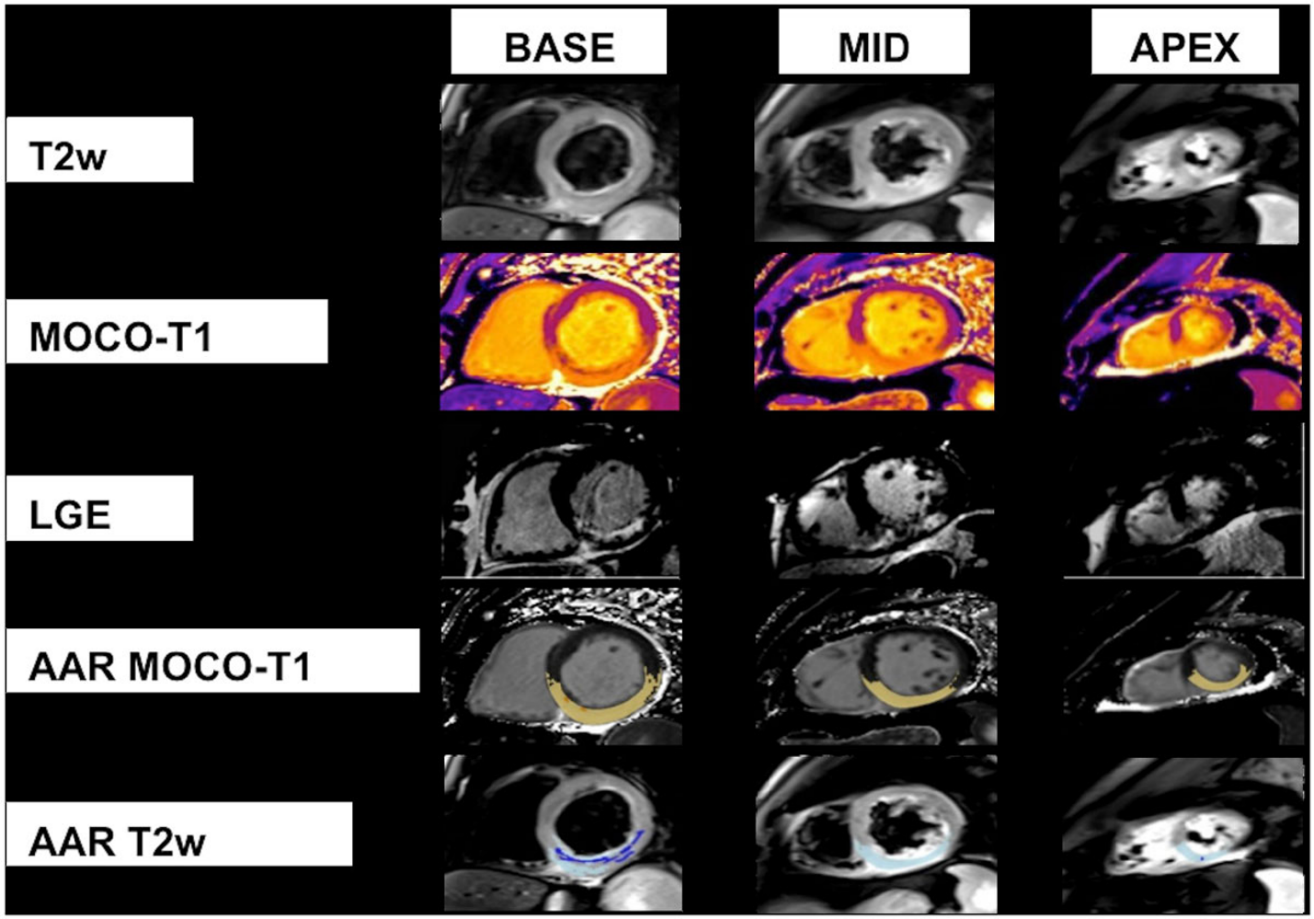
**CMR short axis LV images demonstrating enhancement consistent with myocardial oedema (AAR) by T2w imaging (rows 1 and 5) and by MOCO-T1 (rows 2 and 4) referenced to infarction (row 3)**. *AAR, area-at-risk; CMR, cardiovascular magnetic resonance; LGE, late-gadolinium enhancement; LV, left ventricular; MOCO, motion-corrected; T2w, T2-weighted*

See Table 1 and Figure 1. AAR assessed using T1-mapping and T2w-STIR was not significantly different (p = 0.182) with excellent correlation (p=<0.001) and good agreement, although with wide limits of agreement. However, only 70% of T2w-STIR images were of diagnostic quality and a higher diagnostic imaging rate is achieved with T1-mapping (96%) compared with T2w-STIR.

**Table 1:** Comparison of myocardial oedema assessed using T2w-STIR and T1-mapping

	T2w-STIR	T1-mapping
**Diagnostic quality (n, %)**	39/55 (70.9)	53/55 (96.4)
**AAR, g (mean ± SD)**	35.4 ± 16.0	37.0 ± 16.5
**R (Pearson's Correlation)**	0.91	
**Paired Mean difference (SD)**	1.54 (6.95)	
**95% Limits of agreement**	-12.1 to 15.2	
**CoV (%)**	19.2	
**ICC**	0.95	

AAR, area at risk; CoV; Coefficient of Variation; ICC, Intra-class Correlation Coefficient; SD, standard deviation; T2w-STIR, T2-weighted short tau inversion recovery

## Conclusions

MOCO-T1-mapping is more robust than T2w-STIR for identification of reversible myocardial injury and prediction of functional recovery following STEMI. Furthermore, MOCO-T1 imaging may allow AAR to be accurately determined without long breath holds, often required for the acquisition of oedema imaging, in acutely unwell patients. This requires validation in larger studies and may have implications for sample size calculations in trials using CMR surrogate markers of myocardial injury.

